# Cerebral venous thrombosis revealing an ulcerative colitis

**DOI:** 10.11604/pamj.2016.23.120.9186

**Published:** 2016-03-24

**Authors:** Abdellah Taous, Maha Aït Berri, Taoufik Lamsiah, Brahim Zainoun, Tarik Ziadi, Abdelhadi Rouimi

**Affiliations:** 1 Department of Neurology, Military Hospital Moulay Ismail, Meknes, Morocco; 2 Department of Gastro-Enterology, Military Hospital Moulay Ismail, Meknes, Morocco; 3 Department of Radiology, Military Hospital Moulay Ismail, Meknes, Morocco

**Keywords:** Cerebral venous thrombosis, inflammatory bowel diseases, ulcerative colitis

## Abstract

Cerebral venous thrombosis (CVT) has been reported as an uncommon and devastating complication of ulcerative colitis (UC), with an annual incidence varying between 0,5 to 6,7%. It is suspected to be a consequence of the hypercoagulable state occurring during disease relapse. We report a case of 22-year-old female patient presenting with CVT revealing an UC. Our case raises the awareness among health professionals about the inflammatory bowel diseases (IBD) as a rare etiology of CVT, and signifies the importance of considering antithrombotic prophylaxis in all hospitalised IBD patients, especially those with active disease.

## Introduction

Ulcerative colitis (UC) is an inflammatory bowel disease (IBD) that may have various neurologic manifestations which seem to be more common than previously estimated. There is evidence of an increased incidence of thrombotic complications in patients with ulcerative colitis (UC) and Crohn's disease. However, cerebral vascular involvement is rare and only 1.6% of total cerebral venous thrombotic events are associated with IBD [1, 2]. We report a case of 22-year-old female patient presenting with CVT revealing an UC.

## Patient and observation

**Figure 1:**
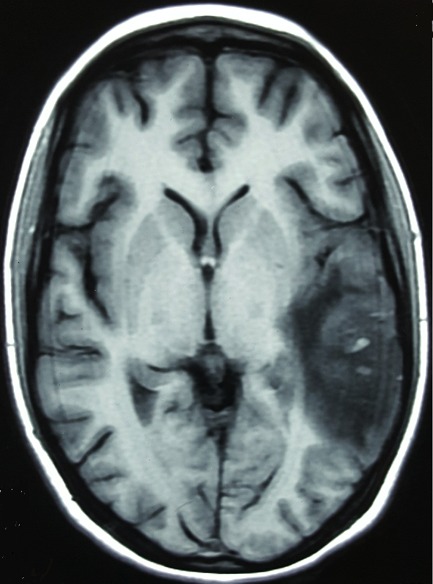
Magnetic resonance imaging. Contrast enhanced Axial T1 image showing a temporoparietal hypointense area with few petechial lesions

**Figure 2:**
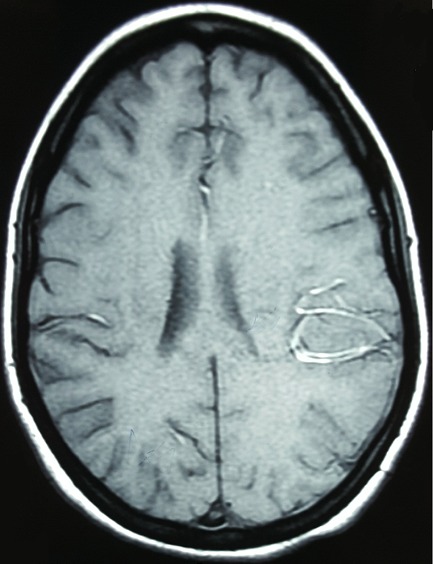
Magnetic resonance imaging. Gadolinium-enhanced axial T1 image showing a meningeal enhancement at the left parietal lobe

**Figure 3:**
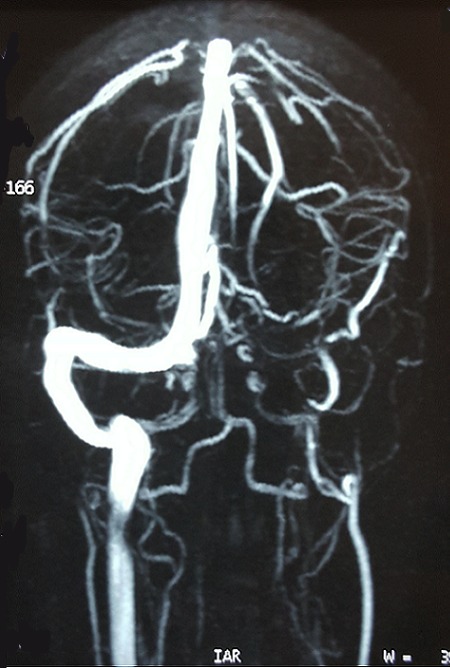
Magnetic resonance venogram image showing abrupt loss of flow related signal at the proximal left transverse sinus continuing distally to involve the left sigmoid

A 22 year-old female was presented to our department with a sudden onset of language disorders. A week earlier, she experienced an intense and diffuse abdominal pain, bloody diarrhea and intermittent vomiting. She had no particular medical history, especially no cardiovascular risk factors. Physical examination revealed a pale, febrile patient at 39 °C, with low blood pressure (90/60 mmHg) and tachycardia at 105 beats per minute. The abdomen was sensitive and on the digital rectal examination the stall was stained with blood. Neurological examination found a Wernicke aphasia without other deficiencies. A contrast-enhanced computed tomography scan of the brain showed a left temporo-parietal venous infarct with incomplete enhancement of left transverse sinus. A cerebral angio-magnetic resonance imaging confirmed the venous infarct and revealed an absence of signal in the left transverse and sigmoid sinus (Figure 1, Figure 2, Figure 3). Laboratory findings showed anemia at 7g/dl, the white blood cell count was elevated at 13000/mm^3^. Erythrocyte sedimentation rate was 90 mm in the first hour. CRP elevated at 103mg/l. Prothrombine time and PTT were normal. Liver function, renal function, serum electrolytes were within normal limits. His prothrombotic workup such as factor V Leiden, protein C and S, factor VIII, and antithrombin III was normal. Antinuclear antibodies and anticardiolipin antibodies were negative and homocysteine levels were normal. In addition, lumbar puncture and stool examination were also normal whereas the sigmoidoscopy completed by a total colonoscopy with biopsies have found a pancolic ulcerative colitis. Taking into account clinical and laboratory criterias of Truelove-Witts (8-10 hemorrhagic motions per day, Hb 7g / dl CRP 103 mg/l) the diagnosis of UC severe flare was retained and the patient was given intravenous pulse steroid therapy (methylprednisolone 40mg / d) in addition to anticoagulation therapy. She was put on maintenance treatment with Acénocoumarol and prednisolone started with 60 mg/day and slowly tapered off over 5 months. We observed a regression of neurological and bowel symptoms after the first week and a complete clinical and biological recovery after three months of follow up.

## Discussion

Inflammatory bowel diseases comprise two major entities: ulcerative colitis and Crohn disease. UC is an idiopathic chronic IBD that is a consequence of complex interaction of environmental factors and genetic susceptibility [3]. It often occurs in patients between the ages of 20 and 30 years, with a second peak between the ages of 70 and 80 years. UC can be regarded as a systemic disease with numerous extraintestinal complications. Neurologic manifestations are protean, rare and particularly severe [4]. There is a high thromboembolic risk in IBD patients with an annual incidence varying between 0,5 [5] to 6,7% [6]. Deep venous thrombosis and pulmonary thromboembolism are the two most common thrombotic complications of UC [7]. CVT has been reported as an uncommon but severe complication of UC and CD, ranging in frequency from 1.3% up to 7.5% of cases yearly depending on the clinical study [8]. Various mechanisms have been postulated for thrombosis in UC which include hypercoagulation (elevated FVIII, fibrinogen, decrease in antithrombin, protein S and protein C), hypofibrinolysis [elevated PAI-1 and lipoprotein (a)], platelet abnormalities, endothelial dysfunction (increased von Willebrand factor), and immunological abnormalities (antiphosphlipid antibodies) [9]. Concomitant causes of CVT were systematically searched for in our patient. There were no risk factors and the prothrombotic workup was negative. Probably in the acute phase of the illness our patient may have had a hypercoagulable state. The clinical presentation of CVT, consisting of headaches, focal signs, seizures, or encephalopathy, and the sites of the venous occlusions are similar to the usual cerebral venous thrombosis. They can occur from 2 months to 17 years after the first attack of IBD. Occasionally, the diagnosis of IBD is established only when CVT occurs [10, 11]. Although IBD may be asymptomatic when the venous thrombosis occurs, almost all patients had biologic markers of inflammation such as elevated leukocyte count, CRP, or ESR. The first-line treatment for CVT is adjusted-dose unfractionated heparin or low-molecular-weight heparin, but its risks should be carefully weighed in light of possible hemorrhagic complications [12]. Endovascular thrombolysis was tried in a few cases, with favorable and safe outcomes [13]. This limited evidence supports the use of approved guidelines for cerebral venous thrombosis management [12, 14] also when associated with IBD. The prognosis is usually good, but a few cases were fatal [10, 11].

## Conclusion

Among the multiple causes of CVT, the IBD must always be in mind. Early diagnosis and management might improve its poor prognosis. Subsequently, prophylactic anti-coagulation must be considered in all patients with IBD, especially in severe flare.
